# Impact of overweight and obesity on disease activity and remission in systemic lupus erythematosus: A systematic review and meta-analysis protocol

**DOI:** 10.1371/journal.pone.0287753

**Published:** 2023-06-29

**Authors:** Marília Cristina Santos de Medeiros, Karla Simone Costa de Souza, Ony Araújo Galdino, Ricardo Ney Cobucci, Adriana Augusto de Rezende

**Affiliations:** 1 Postgraduate Program in Health Sciences, Federal University of Rio Grande do Norte, Natal, Brazil; 2 Department of Clinical and Toxicological Analyses, Federal University of Rio Grande Do Norte, Natal, Brazil; 3 Graduate Program of Biotechnology, Universidade Potiguar (UnP), Natal, RN, Brazil; 4 Graduate Program in Sciences Applied to Women’s Health, Maternidade Escola Januário Cicco (MECJ/ EBSERH), Federal University of Rio Grande do Norte, Natal, Brazil; Endocrinology and Metabolism Population Sciences Institute, Tehran University of Medical Sciences, ISLAMIC REPUBLIC OF IRAN

## Abstract

**Background:**

Systemic lupus erythematosus (SLE) is an autoimmune and inflammatory disease that requires treatment with hydroxychloroquine and glucocorticoids. Glucocorticoids are responsible for adverse effects such as increased weight, which can modify the severity and chronicity of autoimmune pathologies.

**Aim:**

To summarize scientific evidence regarding the impact of overweight and obesity on disease activity and remission in SLE.

**Methods:**

The protocol was developed according to the Preferred Reporting Items for Systematic Review and Meta-analysis Protocol (PRISMA-P) and published in the International Prospective Register of Systematic Reviews database (PROSPERO—CRD42021268217). PubMed, Scopus, Embase, and Google Scholar will be searched for observational studies including adult patients with SLE who were overweight and obese or not, that included disease activity or remission as outcomes. The search is planned for May 2023. Three independent authors will select the eligible articles and extract their data. Subsequently, three authors will independently extract data from each included study using an extraction form created by the researchers. Methodological quality analyses will be performed using the modified Newcastle-Ottawa scale. The results will be presented as a narrative synthesis according to the synthesis without a meta-analysis reporting guideline (SWiM). Meta-analysis will be conducted where appropriate using random-effects models.

**Expected results:**

This review will identify the impact of overweight and obesity on the clinical features of SLE, helping clinicians manage disease activity and remission, both important to optimize disease outcomes and patient quality of life.

## Introduction

Systemic lupus erythematosus (SLE) is a multisystem disease that affects one or more organs, with periods of flare or remission. Its diagnosis is based on clinical evidence of characteristic serological abnormalities, for example antinuclear and more specific autoantibodies, like anti-dsDNA and anti-Sm [1]. Due to its multisystem characteristic, SLE has a wide spectrum of manifestations: since mild manifestations as fatigue, arthralgia, and oral ulcerations, until more severe manifestations: kidney, hematological and neurological diseases [2, 3].

Treatments of flares of SLE are based on the severity of organ(s) involvement using drugs such as hydroxychloroquine or glucocorticoids [1]. The primary aim of the treatment is to achieve remission or low disease activity and prevention flares [1]. Although the use of glucocorticoids is necessary, prolonged use of this medication has adverse effects, such as changes in body composition (weight gain) and lipid profile [3].

Obesity and overweight are identified as risk factors for flare in patients with SLE, by the expression of inflammatory cytokines such as tumor necrosis factor-alpha (TNF-α) and interleukin 6 (IL-6) [4, 5]. In addition, serum lipid levels tend to increase during the active disease course compared to the non-active disease. Thus, it is important to manage obesity because it is a modifiable risk factor and comorbidity in patients with SLE since it may worsen disease activity that can limit functionality and quality of life [6]. Studies of other autoimmune diseases (rheumatoid arthritis and inflammatory bowel disease) have shown that obesity can also negatively affect disease activity [7, 8].

The impact of overweight and obesity on disease activity and remission in SLE remains unclear, and most studies conducted focused on describing the nutritional status of patients with SLE [9] without evaluating the influence of overweight and obesity on disease activity and remission.

Thus, it is important to investigate this association since treatment is aimed at achieving remission of the disease. We aimed to provide a systematic review protocol to summarize the scientific evidence regarding the impact of overweight and obesity on disease activity and remission in SLE.

## Methods

The systematic review protocol follows the recommendations of the Preferred Reporting Items for Systematic Review and Meta-analysis Protocol (PRISMA-P) [10]. The protocol was registered in the International Prospective Register of Systematic Reviews (PROSPERO) (registration number: CRD42021268217). The systematic review will follow the guidelines of the PRISMA [11].

### Ethics

Ethical approval is not required because this review will retrieve publicly available scientific literature. Traditional dissemination strategies will be used, including open-access, peer-reviewed publications, scientific presentations, and reports.

### Inclusion criteria

This systematic review will include observational studies (cross-sectional, cohort, and case-control studies) published until April 2023, which describe the impact of overweight and obesity on disease activity and remission in adults with SLE based on the PECOS elements. No restrictions will be applied to the language or publication period.

### Exclusion criteria

Randomized clinical trials, review articles, reports, and case series will not be included in this review. Studies that assessed other types of lupus or patients aged < 18 years old will also be excluded. Published articles but not peer-reviewed articles will not be included in the review.

### The PECOS strategy

The PECOS strategy used in the review question is shown in Table 1.

**Table 1:** Definition of the structured review question in the form of the acronym PECOS.

Description	Abbreviation	Elements
Population	P	Adults with SLE (>18 years old) with overweight and obesity or not.
Exposure	E	Overweight and obesity
Comparator/control	C	Adults with SLE and adequate body mass index (BMI)
Outcomes	O	Disease activity, remission, inflammatory markers
Study	S	Observational studies (cross-sectional, cohort, and case-control)

Abbreviations: SLE- Systemic lupus erythematosus, BMI- Body mass index

To diagnose SLE, studies should be adopted with the classification criteria for SLE jointly supported by the European League Against Rheumatism and the American College of Rheumatology [12]. To diagnose overweight and obesity, the authors should adopt a reference from the World Health Organization [13], which people with body mass index (BMI) between 25–30 kg/m^2^ are considered with overweight and people with BMI over 30 kg/m^2^ are considered with obesity. We will include studies that evaluate overweight and obese groups measured by body mass index category or visceral fat or body composition (percentage of body fat).

The primary outcomes evaluated in this systematic review are disease activity and remission rates. These outcomes are chosen because measurement of disease activity in SLE is central to clinical research when evaluating clinical outcomes, comparing meaningful differences among SLE patient groups, and assessing disease activity longitudinally in observational and clinical trials [14]. The therapeutic goal of any given treatment intended for chronic use is to induce a clinically relevant reduction in the activity of the disease that is maintained in the long term [15].

Many scales are available for disease activity. Composite disease activity measures are used for overall disease activity, which often reflects multiple organ involvement with many serological abnormalities [16]. We will select studies that used the the main tool to evaluate disease activity, SLE Disease Activity Index (SLEDAI), with scores ranging from 0 to 105 points, and the disease is classified as active when the patient presents above 1 point. Complete clinical remission is defined as the complete absence of disease activity measured by disease activity indices in patients who do not require any ongoing lupus-specific therapy. For patients receiving steroids, including those who are steroid-dependent, the goal of treatment is steroid-free or at least to achieve a low steroid dose to maintain remission [15].

The secondary outcomes are inflammatory markers (C-reactive protein, interleukin 6, leptin, homocysteine, and complement components C3 and C4), and other pro-inflammatory cytokines and chemokines measured in serum/plasma. Studies have shown that increased inflammatory markers are associated with active SLE or flare, indicating that treatment may fail. Few studies have evaluated the effects of overweight and obesity on these inflammatory markers in patients with SLE [17, 18].

### Search strategy

The studies will be obtained through PubMed, Scopus, Embase and Google Scholar databases, looking for the Mesh Terms on title, keywords and abstract. No restrictions will be placed based on languages and year of publication. Reference lists of eligible studies and grey literature will also be screened to identify additional studies. The search strategy used for PubMed is shown in Table 2. The search strategies used in other databases are in S2 File.

**Table 2:** Search strategy for PubMed.

MESH terms	
1	Obesity
2	Overweight
3	Body weight
4	Body mass index
5	Body composition
6	Abdominal fat
7	Visceral fat
8	Obesity, abdominal
9	Body fat distribution
10	Adiposity
11	OR / 1–10
12	Systemic lupus erythematosus
13	Lupus erythematosus, systemic
14	OR / 12–13
15	11 AND 14

### Data collection and analysis

All manuscripts identified after each database search will be screened by three independent reviewers (MCSM, KSCS, and OAG), and duplicates will be removed. The review team will independently read the titles and abstracts based on the inclusion criteria.

The full texts of these potentially eligible studies will be retrieved and taken independently for eligibility by three members of the review team (MCSM, KSCS, and OAG).

**Fig 1:**
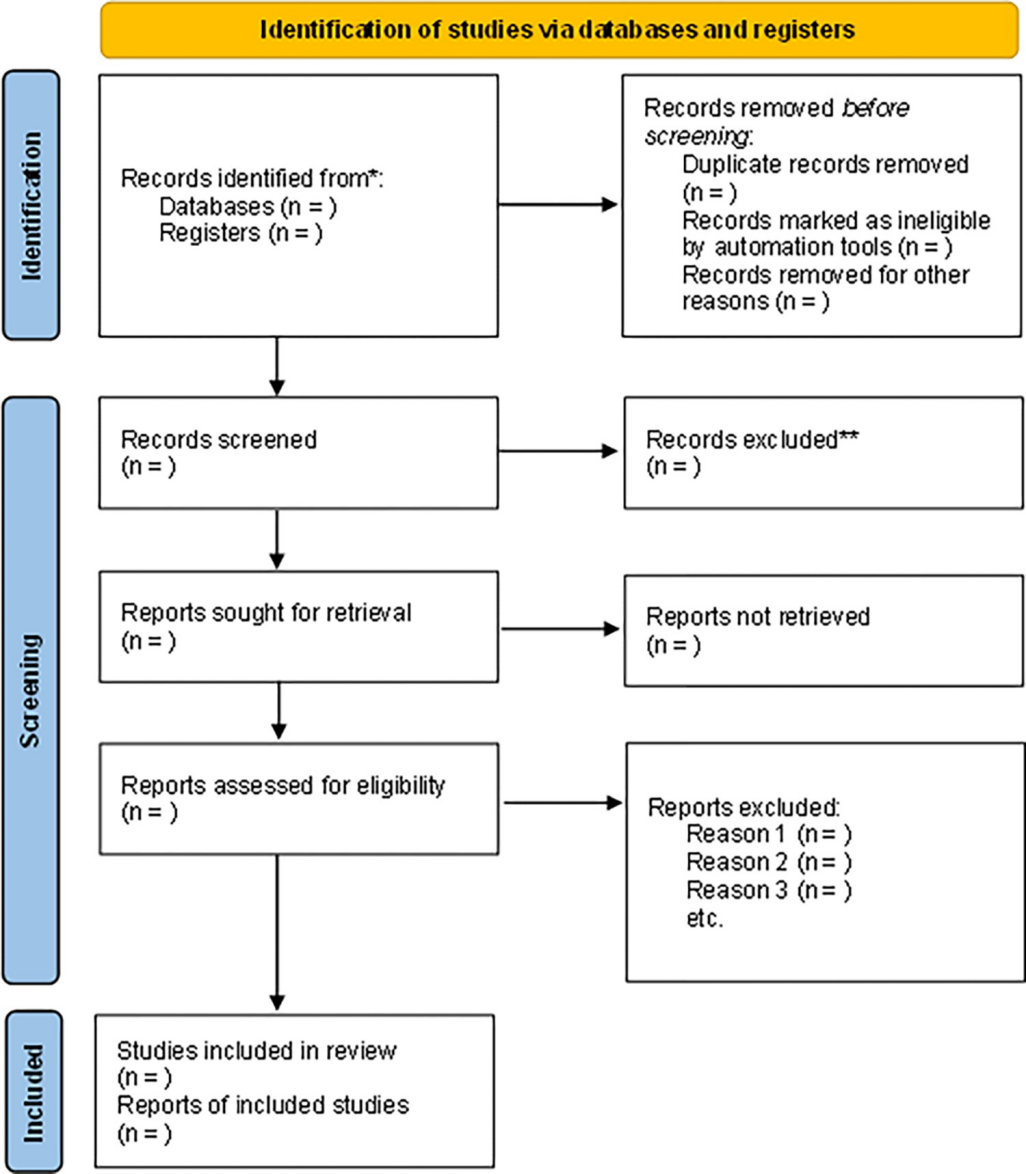
PRISMA flow diagram for systematic review and meta-analysis.

The team will register reasons for excluding studies at all stages of the review. Differences will be resolved using an inter-rater agreement with the Cohen kappa test. If no consensus is reached, a fourth researcher (AAR) will be consulted. Citations will be collected Rayyan QCRI (Rayyan QCRI, Qatar Computing Research Institute, HBKU, Doha, Qatar). The results of the selection or exclusion of studies will be reported using the PRISMA flow diagram, as shown in Fig 1.

The anticipated start date for the selection of articles is April 2023 and the expected completion date is May 2023.

#### Data extraction

Three independent reviewers (MCSM, KSCS, and OAG) will independently extract data from each included study using an extraction form created by the researchers.

The data extracted will include references (author, year of publication), study characteristics (design, sample, and country), and participant characteristics (age, sex, body mass index category, body composition [percentage of body fat], waist-hip ratio, disease activity, remission, and inflammatory markers). The data extraction form is available in S3 File.

### Addressing missing data

In the case of missing data, the review team will contact the respective authors of the articles by e-mail. If information is not received, the data will be excluded from our analysis and discussed in the respective section.

### Quality assessment of the included studies

A qualitative synthesis will be developed regarding the impact of overweight and obesity on disease activity and remission in patients with SLE. The data will be organized into tables and descriptively analyzed.

The modified Newcastle–Ottawa Scale [19] tool will be used to assess the quality of the studies. Three reviewers (MCSM, KSCS, and OAG), will independently assess the quality of the included studies. Quality will be rated as high or low considering the total number of points received: ≥ 4 for good quality and <4 for low quality.

Publication bias will be evaluated if more than 10 studies have been included with a funnel plot. In addition, Egger’s weighted correlation and Begg’s regression intercept at a 5% significance level will be conducted [20].

### Assessment of heterogeneity

#### Measures of effect size

We will use the ‘meta’ package of Stata to enter the data. We will extract or calculate the odds ratio (OR) and 95% confidence interval (CI) for each study in the presence of dichotomous results. When there is heterogeneity (I^2^ ≥50%), a random-effect model will combine the studies to calculate the OR and 95% CI.

We will use the χ^2^ test to evaluate the study outcomes (significance level of p<0.1). Heterogeneity will be evaluated according to the Cochrane Handbook [21] criteria using the I^2^ statistic. We consider that a value of 0% indicates a lack of heterogeneity in studies; ≥50% values indicate considerable heterogeneity. Meta-regression analyses will be performed to investigate the possible causes for different effect sizes and to deal with considerable heterogeneity across studies. It is essential to mention that this evaluation will be executed if the meta-analysis’ (MA) achievement is appropriate.

The extracted data will be quantitatively pooled and analyzed in the ‘meta’ package of Stata statistical software version 16.0 (StataCorp, College Station, Texas, USA). Statistical significance determination will materialize at a p-value of <0,05.

#### Data synthesis

A quantitative synthesis (meta-analysis) will be performed in ‘meta’ package of Stata software using the inverse variance method with the random effects model if there is more than 50% heterogeneity between the studies [22]. In cases where the data will be insufficient to calculate an effect estimate, a narrative synthesis will be created, describing the direction and size of the effects, along with any reported accuracy measure.

#### Subgroup and sensitivity analysis

We plan to perform subgroup analyses, if data are available, by age, gender and body mass index category (overweight, obesity classes I, II, and III) [13]. Sensitivity analysis will be conducted to verify possible sources of heterogeneity, removing one study at a time and verifying if there is a considerable change in the OR and 95% CI. Sensitivity analysis will be performed, excluding low-quality studies. When the effect estimates of the primary and sensitivity analyses are significantly different, an adjusted sensitivity analysis will be performed.

### Grading quality of evidence

#### Assessment of certainty of the evidence

We will use the Grading of Recommendations Assessment, Development, and Evaluation Working Group methodology [23] to analyze the evidence for all outcomes, classifying the evidence as high, moderate, low, or very low.

## Discussion

Obesity is associated with complications in diverse autoimmune diseases and is very common in patients with inflammatory rheumatic diseases, of which 27% and 37% of patients with rheumatoid and psoriatic arthritis, respectively have a body mass index ≥30 kg/m^2^ [24]. In patients with SLE, the frequency of obesity is similar or higher than that in the general population, with a prevalence ranging from 28% to 50%, and it has been implicated as a risk factor for exacerbation of clinical manifestations of disease and inflammatory processes [25]. It may be attributed because one of the characteristics of obesity is a state of chronic low-grade inflammation. This condition induces the production of pro-inflammatory cytokines such as TNF-α, IL-6, and adipokynes such as leptin, produced by white adipose tissue [26]. A meta-analysis demonstrated that patients with SLE have higher levels of leptin compared to control group. Leptin is responsible for activating monocytes/macrophages to release proinflammatory cytokines such as TNF-a and IL-6, activates monocytes, dendritic cells, and macrophages, and stimulates the differentiation of T lymphocytes into Th1 phenotype. [27] Furthermore, oxidative stress caused by obesity can lead inflammation through the transition of adipose tissue macrophages from M2 to M1, leading T cells recruitment [28]. Another study supported the hypothesis that obesity promotes disease activity in psoriatic arthritis [29]. In inflammatory rheumatic diseases, it has been hypothesized that patients with obesity tend to have low response rates for antirheumatic drugs (conventional synthetic disease-modifying antirheumatic drugs) and biological drugs [24]. A study with patients with rheumatic diseases suggests that patients with obesity may require more time of treatment to achieve minimal activity disease [30]. But it is necessary to understand if patients with SLE have the same response.

Studies on the impact of obesity on disease activity and remission in SLE are scarce and generally focus on describing the nutritional status of patients with SLE [9]. Therefore, this study will contribute to providing objective evidence about the impact of obesity on disease activity and remission in SLE, since obesity is a modifiable risk factor responsible for increased disease activity and an altered lipid profile. Excess adiposity is associated with a greater symptom burden, such as cardiovascular risk factors (hypertension, dyslipidemia), atherosclerosis, and metabolic syndrome, and they impact the quality of life, a neglected area by some clinicians. This evidence may help clinicians offer the best treatment for patients, optimize disease outcomes, and improve their quality of life [4, 6].

This systematic review may have limitations, as it will include only observational studies. If most of the included studies are cross-sectional or case-control, it would preclude a reliable assessment of the causal relationship between obesity and disease activity and remission in SLE. Other limitations regarding study characteristics, such as sample size and the limited number of studies, can also influence the validity and reliability of the findings. Furthermore, BMI, one of the methods used to measure obesity, does not distinguish fat and leans mass. Despite these limitations, the review team will use a rigorous process to identify and analyze studies that respond to this systematic review. The purpose of the protocol was justified because systematic review findings may represent quality evidence proving that overweight and obesity impede treatment of SLE in adults. A better understanding of the impact of overweight and obesity on disease activity and remission in SLE is essential to optimize disease outcomes and offer patients a better quality of life.

## Supporting information

S1 File Prisma P 2015 checklist. (DOCX)

S2 File Search strategies applied for other databases. (DOCX)

S3 File Data extraction form. (DOCX)
