# Supplementary material for: Impact of overweight and obesity on disease activity and remission in systemic lupus erythematosus: A systematic review and meta-analysis protocol
Source: PLoS One. 2023 Jun 29;18(6):e0287753. doi: 10.1371/journal.pone.0287753 (PMC10309980; doi:10.1371/journal.pone.0287753)
Supplement: S2 File — (DOCX) [file pone.0287753.s002.docx]

Search strategies applied for other databases

1. PUBMED, SCOPUS, EMBASE

| MESH terms | |
| --- | --- |
| 1 | Obesity |
| 2 | Overweight |
| 3 | Body weight |
| 4 | Body mass index |
| 5 | Body composition |
| 6 | Abdominal fat |
| 7 | Visceral fat |
| 8 | Obesity, abdominal |
| 9 | Body fat distribution |
| 10 | Adiposity |
| 11 | OR / 1 – 10 |
| 12 | Systemic lupus erythematosus |
| 13 | Lupus erythematosus, systemic |
| 14 | OR / 12 - 13 |
| 15 | 1. AND 14 |

1. GOOGLE SCHOLAR

(obesity OR overweight OR "body weight" OR "body mass index" OR "body composition" OR "abdominal fat" OR “visceral fat” OR “obesity, abdominal” OR “body fat distribution” OR adiposity) AND (“systemic lupus erythematosus" OR “lupus erythematosus, systemic”)
