# Supplementary material for: Impact of overweight and obesity on disease activity and remission in systemic lupus erythematosus: A systematic review and meta-analysis protocol
Source: PLoS One. 2023 Jun 29;18(6):e0287753. doi: 10.1371/journal.pone.0287753 (PMC10309980; doi:10.1371/journal.pone.0287753)
Supplement: S3 File — (DOCX) [file pone.0287753.s003.docx]

**Data extraction form**

| Author/year of publication | Study design | Sample | Country | Age | Sex | BMI | % fat mass | Waist-hip ratio | Disease activity | Remission | Inflammatory markers |
| --- | --- | --- | --- | --- | --- | --- | --- | --- | --- | --- | --- |
|  |  |  |  |  |  |  |  |  |  |  |  |
|  |  |  |  |  |  |  |  |  |  |  |  |
|  |  |  |  |  |  |  |  |  |  |  |  |
